# Diffusive drug delivery in the brain extracellular space from a cellular scale microtube

**DOI:** 10.1557/s43579-022-00247-9

**Published:** 2022-09-19

**Authors:** Marie-Joe Störi, Pelumi W. Oluwasanya, Christopher M. Proctor

**Affiliations:** Engineering Department, University of Cambridge, Cambridge, UK

**Keywords:** Diffusion, Bioelectronic, Fluidics, Simulation

## Abstract

The effectiveness of state-of-the-art systemic treatments for neurological disorders is hampered not only by the difficulty in crossing the blood brain barrier but also off-target drug interactions. In this study, a delivery method is simulated for a novel U-shaped microtube locally infusing drugs directly into the extracellular space of the brain and relying on diffusion as a transport mechanism. The influence of flow rate, drug properties and device geometry are investigated. It is anticipated that these findings will accelerate progress on both developmental and applied drug delivery and materials research.

**Graphical abstract:**

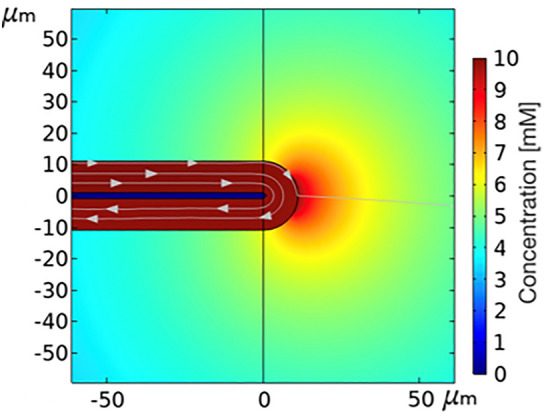

**Supplementary Information:**

The online version contains supplementary material available at 10.1557/s43579-022-00247-9.

## Introduction

Treating neurological disorders such as Parkinson’s disease or gliomas using local drug delivery methods is of high interest as it could lead to a considerable improvement. First, it allows to directly target a specific tissue associated with a given condition leading to more effective therapy with limited side effects. Second, it has the potential to circumvent natural barriers like the blood–brain barrier (BBB) or the blood-cerebrospinal fluid barrier.^[1]^ Indeed, it is estimated that the BBB blocks approximately 98% of small-molecule drugs and $\sim$ 100% of large-molecule drugs,^[2]^ thus a method that avoids this barrier has a huge benefit. Local delivery also allows for a higher concentration of drug to be administered safely compared to systemic delivery.

Many studies have been conducted in the last few decades to develop a local delivery method in the brain. Convection-enhanced delivery (CED) is based on drug injection under high pressure using an intracranial needle or a catheter. The pressure allows to achieve a distance of drug transport of several millimetres and the method has shown some promising results to treat diseases such as malignant glioma, Parkinson’s disease or Alzheimer’s disease.^[3,4]^ However, CED is facing several challenges. The needle used in CED can have a diameter of a few hundreds of micrometres to a few millimetres triggering foreign body response and making it prone to reflux.^[5]^ Furthermore, by using a convection-driven delivery, a pressure is applied which may damage the brain and cause edema.^[6]^ One solution is electrophoretic drug delivery which allows a dry delivery by letting only the drug molecules pass through a membrane, into the brain. But this method is limited to certain applications as the drug needs to be small and charged and it is a challenge to deliver a high amount of drug.^[7]^ Another approach is retrodialysis, also known as reverse microdialysis, in which drug molecules diffuse across a microporous membrane. Such interventions typically use probes designed for microdialysis with sampling/delivery areas on millimetre to centimetre length scales.^[20–22]^

Reported drug delivery implants to date have also been relatively large and stiff compared to the cells and tissues they are targeting.^[7,8]^ This mismatch in geometry and mechanical properties is well known to invoke foreign body reactions and glial scarring which in turn may further limit the implants’ capacity for efficacious drug delivery. Recent advances in flexible and soft materials, thin-film microfabrication, and device innovations have enabled cellular scale neural recording and stimulation devices,^[8,9]^ opening the door to a future class of similarly scaled implants for local drug delivery.

**Figure 1:**
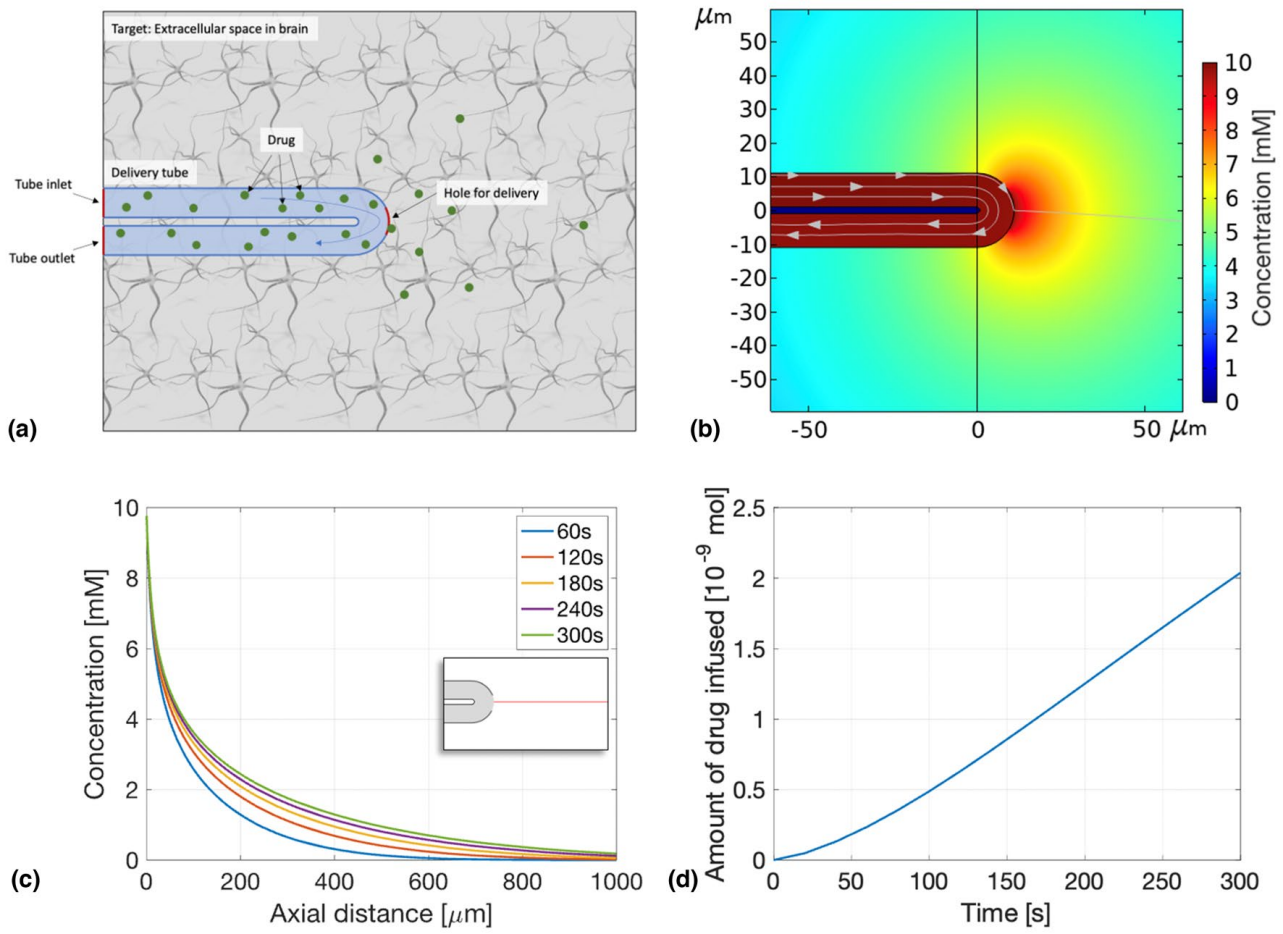
(a) A schematic showing the components of the computational model (not to scale). Results of the time-dependent simulation: (b) Spatial distribution of free drug concentration in the extracellular space of the brain with streamlines showing the total flux. Picture taken after 60 s of simulation. (c) Concentration profile for several time points along axial distance. (d) Amount of diluted species in the brain as a function of time.

Here, we propose a minimally-invasive device to deliver drug in the brain at a cellular-scale using mainly diffusion as a transport mechanism. This device can deliver any type of drug and its small size and flexibility should minimize the foreign body response. The potential applications for this device include delivery of neurotransmitters to study the brain behaviour, and treatment of diseases such as epilepsy and Parkinson’s disease.^[7,10]^ In order to achieve a diffusion-driven delivery, a U-shaped flexible microtube model was designed with both inlet and outlet outside the brain and a small hole in the middle of the tube to allow the drug to diffuse into the extracellular space. A schematic of this model can be seen in Fig. 1(a). An important advantage of a small and diffusion-driven delivery system is the reduction in pressure applied to the brain as well as the footprint of the needle itself which may, in combination lead to lower foreign body response, less reflux and prevent edemas.^[11]^

In the present study, a computational simulation has been conducted to elucidate three main points. The first is to observe how the drug will be delivered by observing its concentration in the brain over time. The second is to see if it is possible to minimize the pressure applied on the brain to achieve a mainly diffusion-driven delivery. Indeed, we would like to achieve a submillimeter scale delivery that is primarily driven by the concentration gradient rather than a pressure-gradient. Lastly, we aim to observe the impact on the delivery process of using different drugs or different sizes or numbers of holes (ie. varying device geometry).

## Materials and methods

The simulations for this study were done using COMSOL Multiphysics®. All the simulations are time-dependent and in 2D. It was estimated that a 2D simulation would allow us to understand the transport mechanisms involved in the device and to observe the influence of the different parameters. It is thus an acceptable approximation for this study.

### Mathematical model

The brain is very densely packed with cells whether we examine grey matter or white matter. The transport of molecules occurs mainly in the space in between the cells, called the extracellular space (ECS). This space accounts for approximately 20% of the total brain volume^[12]^ and can be described as a foam in which the cells correspond to the gaseous phase and the ECS represents the water phase.^[13]^ Given that the ECS is orders of magnitude lower than the distance travelled by drug, the brain can be treated as a porous media and Darcy’s law is thus applied:

Laminar flow:

1 $$\begin{gathered} \frac{1}{\alpha }\rho \frac{\partial \mathbf{u}}{\partial t}+\frac{1}{\alpha }\rho \left(\mathbf{u}\cdot \nabla \right)\mathbf{u}\frac{1}{\alpha }=\nabla \cdot \left[-p\mathbf{I}+\mathbf{K}\right]-\frac{\mu }{\kappa }\mathbf{u} \hfill \\ \rho \nabla \cdot \mathbf{u}=0 \hfill \\ \mathbf{K}= \mu \frac{1}{\alpha }\left(\nabla \mathbf{u}+{\left(\nabla \mathbf{u}\right)}^{T}\right)-\frac{2}{3}\mu \frac{1}{\alpha }\left(\nabla \cdot \mathbf{u}\right)\mathbf{I} \hfill \\ \end{gathered}$$

With $\alpha$ the porosity of the brain, $\rho$ the density, **u** the velocity vector, p the pressure, $\mu$ the dynamic viscosity and $\kappa$ the permeability.

The drug concentration in time is ruled by a modified diffusion equation^[12]^:

Transport of diluted species:

2 $$\frac{\partial C}{\partial t}={D}^{*}{\nabla }^{2}C+\frac{Q}{\alpha }-\mathbf{u}^{{*}}\nabla C-\frac{f(C)}{\alpha }$$

The first term on the left represents how the concentration, $C$, changes in time at a given location. The second term is the diffusion term with ${D}^{*}=D/{\lambda }^{2}$. The free diffusion coefficient $D$ is reduced by the parameter $\lambda$ called tortuosity, which takes into account the geometry, the dead-spaces, the obstructions and the bindings happening inside the ECS. The third term of the equation is the introduction term, this term is equal to zero here as the introduction is included in the boundary conditions of the simulation. The fourth term represents the convection, driven by a corrected velocity vector and the last term incorporates loss, clearance and uptake. Indeed, the diffusing molecules can be permanently removed from the ECS through the BBB, by entering a cell, by binding to receptors or by enzymatic degradation. These processes are often proportional to the concentration and thus $f\left(C\right)= {k}_{\text{elim}}\cdot C$ with ${k}_{\text{elim}}$ the elimination rate.

The two transport mechanisms described in these equations are diffusion and convection. Diffusion is driven by a concentration gradient and convection is driven by a pressure gradient.

### Model geometry

The optimal width of the tube was chosen to be 10 μm, to sufficiently reduce the device footprint while still delivering a significant amount of drug. The distance from the tube inlet to the U-turn and from the U-turn to the outlet is set to 1 cm, resulting in a total tube length of 2 cm. The size of the hole is set to 10 μm but the impact of smaller (5 μm) and bigger (15 μm) hole sizes was studied and presented in the last simulation of this paper. The effect of having several holes is also assessed by implementing 2 and 3 holes in the tube. In the case of 2 holes, they are placed at + 45° and − 45° around the middle horizontal axis. In the case of 3 holes, they are placed at + 90°, 0° and − 90° around the same axis.

In order to avoid any influence of border effect of the simulation model, the brain is defined as a larger surface area than the striking distance of any drug in the time scale studied. Thus, a circle of 20 mm in radius has been chosen.

### Model parameters and assumptions

The inside of the tube was assumed to be filled with water and brain tissue was treated as a porous media. Other material parameters needed for the simulation were taken from Zhan et al.^[14]^. These parameters are shown in Table S1 in the Supporting Information. Parameters set by the user or dependent on the drug delivered are shown in Table S2 in the Supporting Information and described below.

#### Flow rate

The flow rate used in this study is 3 nL/min. The impact of different flow rates is assessed for rates ranging from 1 to 7 nL/min.

#### Pressure

The pressure in the brain, also called the intracranial pressure (ICP), varies a lot. It depends on several aspects as the age, the body posture, and clinical condition and of course it is different whether we look at a human or at a certain animal.^[15]^ For this study, an averaged ICP found in rats was chosen: ${P}_{\text{ICP}} = 536$ Pa. However, the whole study can be easily translated to any other ICP. The ICP is measured as a relative pressure to the atmospheric pressure, thus the pressure at the outlet of the tube is set to zero.

#### Diffusion coefficient

The free diffusion coefficient is dependent on the molecule. It has been shown that parameters such as the size, the shape and the flexibility have an impact, and it is thus difficult to predict the actual diffusion coefficient without measuring it. Furthermore, the diffusion coefficient in the ECS is anisotropic and depends on the location in the brain.^[16]^ For this study, a range of different diffusion coefficients in ECS have been tested but they are assumed to be isotropic and constant over all the simulated brain tissue. The range assessed is ${D}^{*}=\left[1\times {10}^{-11}{\text{ to }} 1\times {10}^{-9}\right]$ m^2^/s including smaller molecules like ions ${(D}^{*}=1-10\times {10}^{-10})$ m^2^/s^[17]^ and larger molecules like proteins ${(D}^{*}=1-10\times {10}^{-11})$ m^2^/s.^[18]^ For simplicity, the free diffusion coefficient is used and calculated using an averaged tortuosity $\overline{\lambda }=\sqrt{D/{D}^{*}}=2$.

#### Drug concentration

The concentration of drug injected in the tube is called here the drug concentration and denoted by ${C}_{\text{d}}$. The influence of this parameter on the diffusion process is investigated. However, for the rest of the simulations, ${C}_{\text{d}}$ is set to 10 mM. In experiments, this number is set by the user but also limited by the solubility of the drug used.

#### Elimination rate

The elimination rate is also molecule dependent. It can be calculated in different ways. For example, Zhan et al.^[14]^ have defined it by combining the elimination rates due to drainage and due to degradation or metabolism. Wolak et al.^[1]^ calculated it using the efflux half-time of the molecule. In this study, a number of elimination rates have been tested from ${k}_{\text{elim}}=1\times {10}^{-4}$ to ${k}_{\text{elim}}=1\times {10}^{-2}$. However, for the other simulations, the elimination rate is set to 0 to remove the influence of this parameter completely and better understand the transport mechanisms.

#### Simulation parameters

The boundaries of the brain are set to the ICP and have a no flux condition. The walls of the tube are set to a no slip and a no flux condition. The outlet of the tube is set to the atmospheric pressure, which in this case is 0 in relative pressures. The mesh is coarse but with a user-defined maximum element size of 2 μm on the curvature (the *U*) of the tube.

## Results

### Concentration profile and time-dependency

The result of the simulation with a 3 nL/min flow rate is shown in Fig. 1. The concentration has a circular distribution around the delivery site [Fig. 1(b)], as expected with an isotropic diffusion coefficient. Also, most of the drug continues along the tube without any disturbance (shown by the streamlines—grey arrows), only the drug far out in the tube diffuses through the hole into the ECS. The concentration profile along a straight horizontal line in the ECS is depicted in Fig. 1(c). The x-axis is the distance to the hole, called the axial distance (0 being the limit between the tube and the ECS). The profile follows an exponential curve and increases with time. No uptake, loss or clearance is included in this initial simulation, thus the drug concentration inside the brain continues to increase. The time step between each line is constant, however, the increase of the concentration profile gets smaller i.e. the difference between the profiles from 1 to 2 min is bigger than the increase from 4 to 5 min. The increase in concentration in the brain can also be observed in Fig. 1(d), which shows the amount of drug in the ECS in time. The amount starts to grow slowly but after approximately 60 s, it reaches a linear regime. This regime continues until the boundaries of the brain domain are reached, in which case the simulations do not depict the reality as no realistic boarders were implemented.

### Effect of the flow rate on the transport mechanism

**Figure 2:**
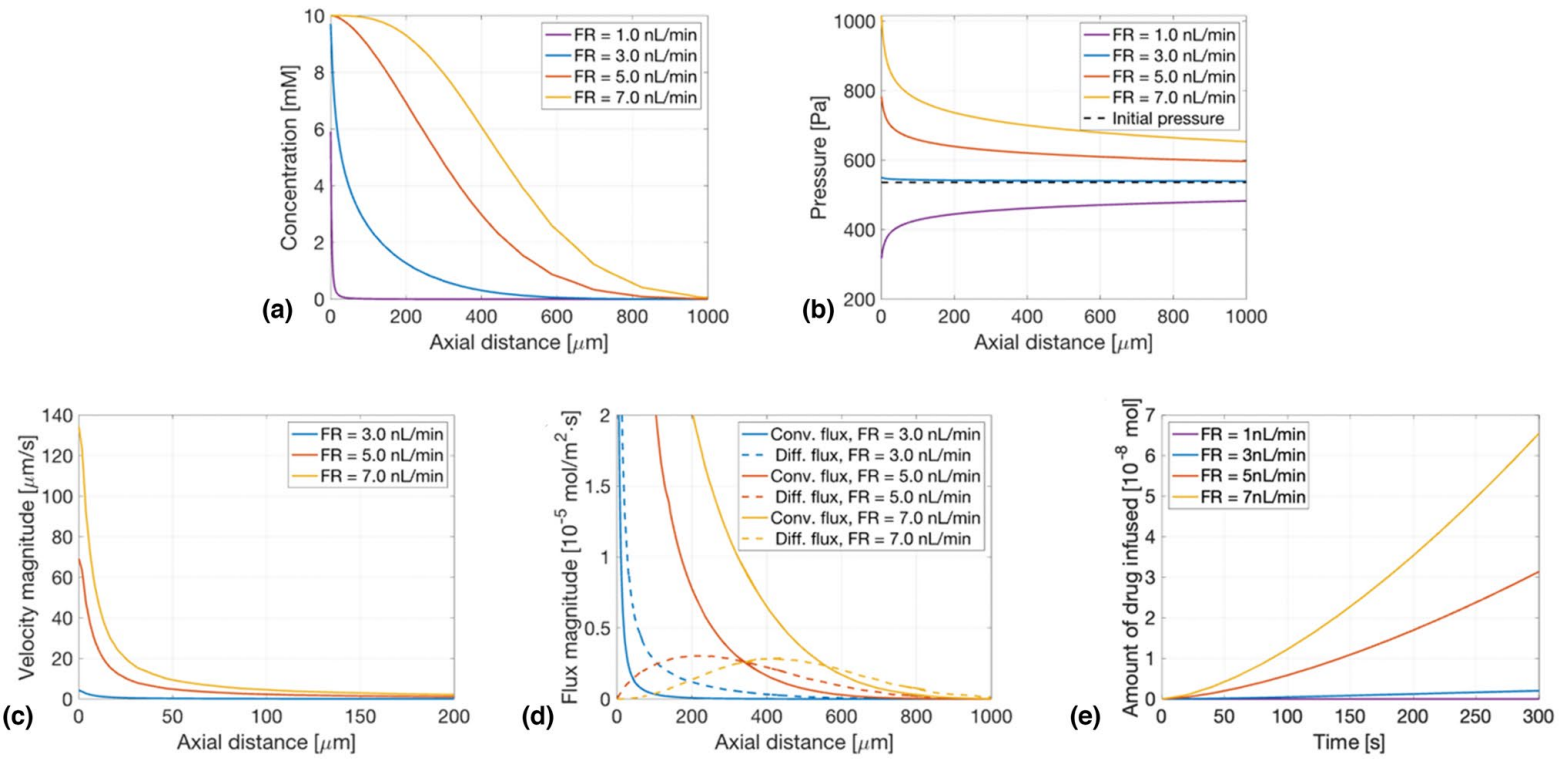
Comparison of profile along axial distance for different flow rates (FR): 1.0 nL/min, 3.0 nL/min, 5.0 nL/min and 7.0 nL/min after 60 s. (a) Concentration profile. (b) Velocity magnitude profile. (c) Pressure profile. (d) Diffusive and convective flux magnitude. (e) Amount of drug infused into the brain for 60 s.

The pressure applied on the brain can be influenced by many different parameters such as the geometry of the tube and the viscosity of the liquid injected. For example, decreasing the tube width while keeping the same flow rate, will increase the applied pressure. However, in this study, the size of the tube is fixed and only the flow rate set at the inlet of the tube is changed. The flow rate is optimized to balance between too much pressure resulting in convection-driven transport and too low pressure inducing backflow. In Fig. 2, concentration, pressure, velocity magnitude and flux magnitude profiles are plotted for four different flow rates along the same horizontal line as in Fig. 1(c).

Figure 2(a) shows that the concentration profiles change with changing flow rates. Indeed, at 3 nL/min, the profile has an exponential shape, but for 5 nL/min or 7 nL/min, the profile becomes like an inverse S-shaped curve. These different concentration profiles can be explained by looking at the pressure [Fig. 2(b)]. At 3 nL/min, the pressure in the brain stays almost exactly constant and is only a few Pascals higher than the pressure initially in the brain (black dashed line). For higher flow rates, the pressure is increased by a few hundreds of Pascals near the hole, multiplying the ICP by 2 just by injecting 7 nL/min into the tube. Similar profiles can be seen when looking at the velocity magnitude [Fig. 2(c)]. Indeed, a pressure gradient induces a velocity and moves the drug towards the lower pressure, this transport mechanism is convection. This can be confirmed by looking at Fig. 2(d), where the convective flux magnitude is compared with the diffusive flux magnitude.

One can see that for higher flow rates such as 5 nL/min or 7 nL/min, the convective flux is higher than the diffusive flux which makes sense as there is a high pressure gradient. For 3 nL/min, the diffusive flux magnitude is higher than the convective one, due to the very low pressure gradient. This explains the difference in the concentration profiles as it can be proven mathematically that a convection-driven transport is characterised by an inverse S-shaped concentration profile, while a diffusion-driven transport has an exponential-like profile. When decreasing the flow rate even more (1 nL/min), the pressure inside the tube gets smaller than the pressure in the brain which creates a backflow and only a very small amount of drug is able to diffuse against this flow and enter the brain [see purple line in Fig. 2(a), (b)]. The last plot of Fig. 2 shows the total amount of drug inside the brain in time. As expected, increasing the flow rate increases the amount of drug in the brain but the shape of the profiles remains the same.

### Impact of drug-dependent parameters on the amount of drug delivered in time

**Figure 3:**
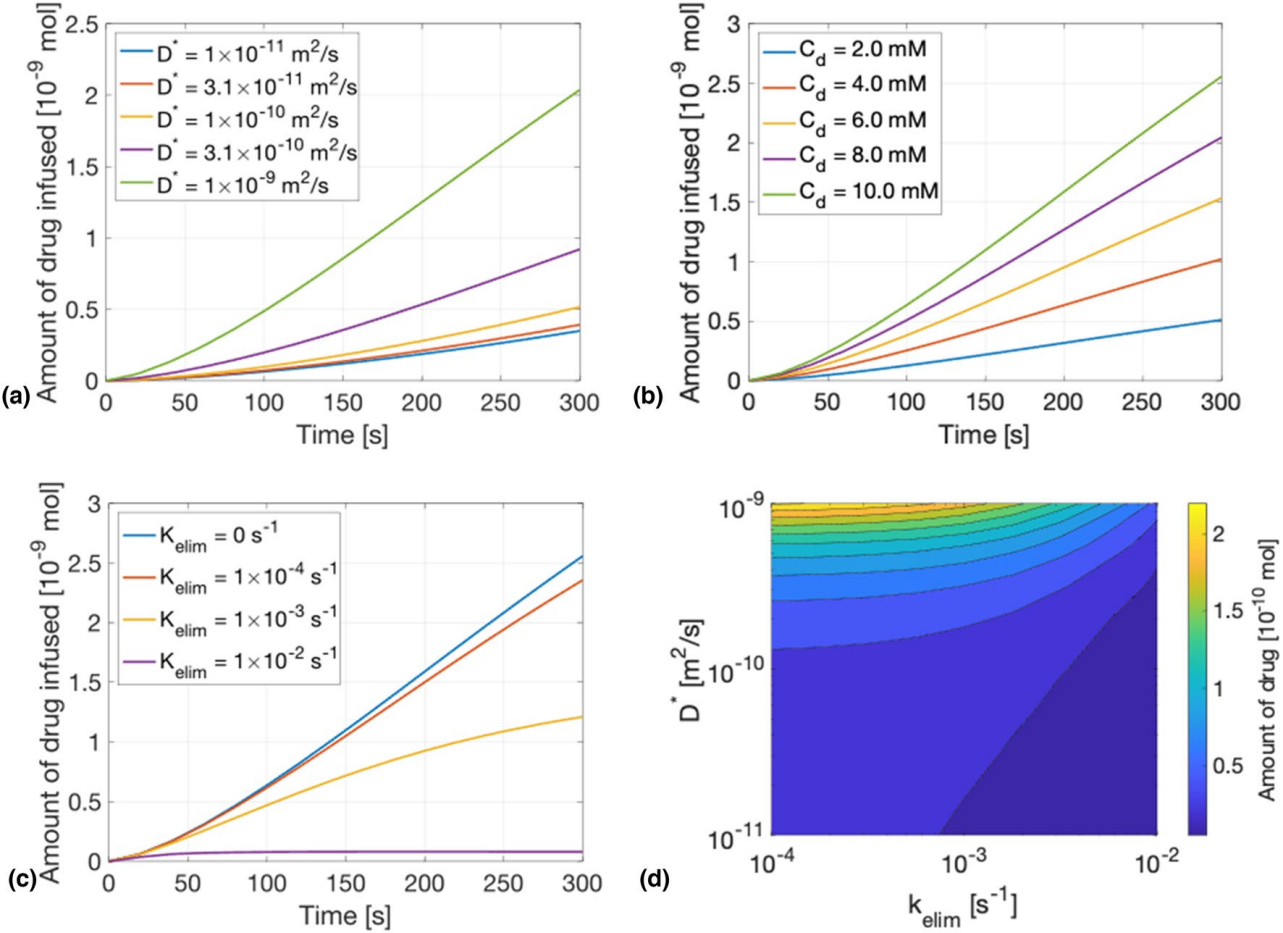
Amount of drug in the brain in function of time with change in (a) Diffusion coefficient. (b) Injected concentration. (c) Elimination rate. (d) Contour plot showing the amount of drug infused depending on the diffusion coefficient and the elimination rate for a drug concentration of 10 mM.

The third part of this research was aimed at understanding the influence of different parameters on the delivery process. These parameters are compared by plotting the amount of drug infused in the brain with time. The diffusion coefficient (${D}^{*}$) is varied from $1\times {10}^{-11}$ to $1\times {10}^{-9}$. The amount of drug delivered increased with the diffusion coefficient [Fig. 3(a)]. From equation Eq. 2, the diffusion term is directly proportional to the diffusion coefficient, meaning the higher the diffusion coefficient, the faster the diffusion process. Nonetheless, multiplying the ${D}^{*}$ by $10$ from $1\times {10}^{-11}$ to $1\times {10}^{-10}$ m^2^/s makes a much smaller difference in the amount of drug infused than increasing ${D}^{*}$ from $1\times {10}^{-10}$ to $1\times {10}^{-9}$m^2^/s. The shape of the curve stays the same for all the different coefficients.

Next, the effect of the drug concentration (${C}_{\text{d}}$) is investigated. As one can observe in Fig. 3(b), the total amount of drug in the brain is linearly proportional to the concentration of drug inside the tube. When the concentration is multiplied by 2 (for example from 2 to 4 mM), so is the total amount of drug infused.

The last parameter swept is the elimination rate, a drug-dependent parameter. One can observe in Fig. 3(c) that the amount of drug infused does not increase linearly anymore but converges to a steady state. This steady state is achieved when the same amount of drug is removed by uptake, loss or clearance as the amount delivered from the tube into the brain. A small elimination rate (${k}_{\text{elim}}=1\times {10}^{-4}{s}^{-1})$ makes almost no difference on the amount of drug, however, increasing it $10$ times or $100$ times decreases a lot the concentration in the brain. Figure 3(d) is a contour plot showing the amount of drug infused after 60 s of simulation while sweeping the diffusion coefficient and the elimination rate simultaneously. The highest amount of drug is achieved by having a high diffusion coefficient and a small elimination rate, while a small amount of drug is achieved by having a small diffusion coefficient and a high elimination rate.

### Impact of size and number of holes on the amount of drug delivered in time

**Figure 4:**
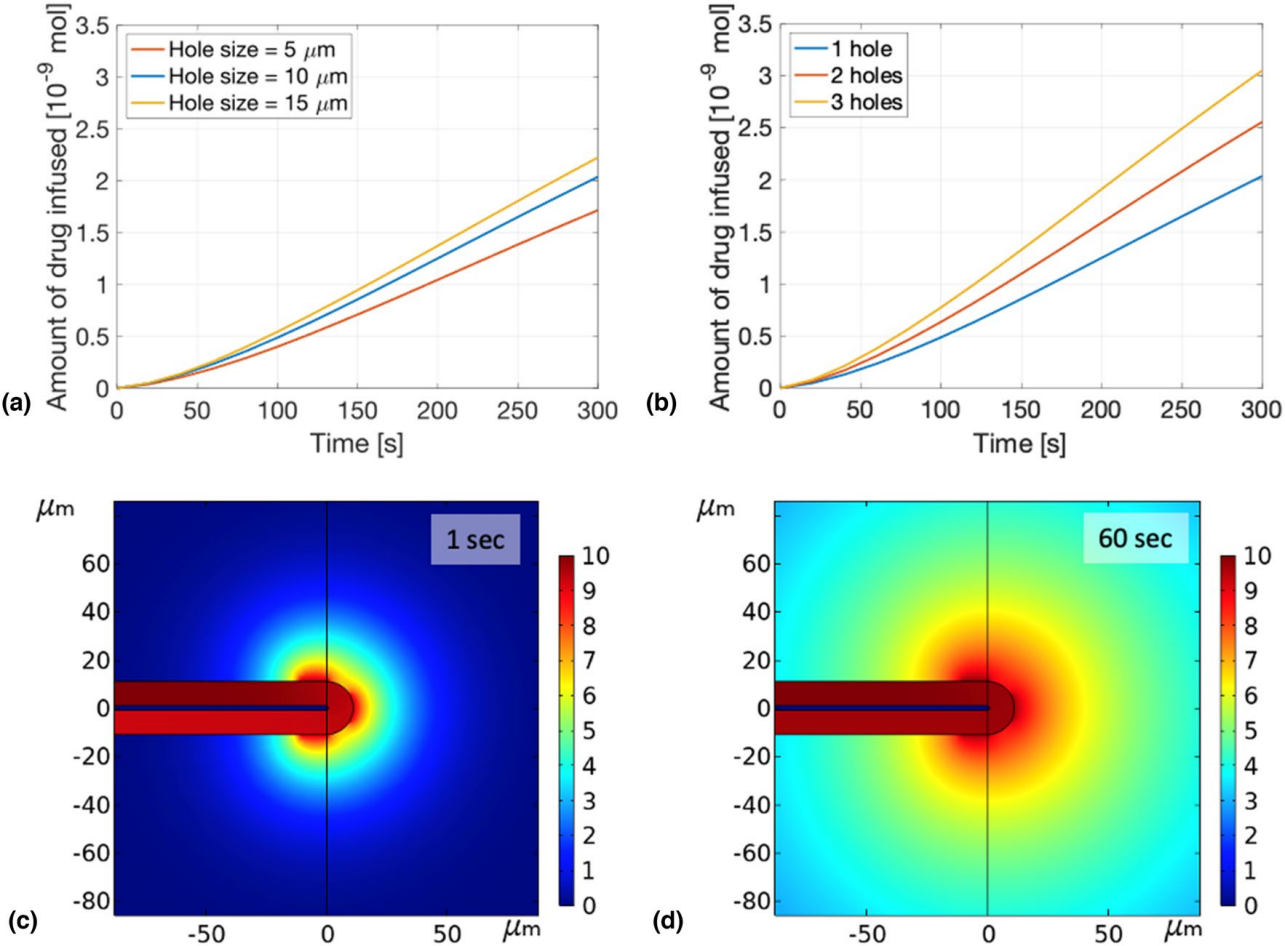
Influence of the hole geometry on the infusion. (a) Amount of drug in the brain in function of time for 3 different hole sizes. (b) Amount of drug in the brain in function of time for 1, 2 and 3 holes. (c) Spatial distribution of drug after 1 s for a tube with 3 holes. (d) Spatial distribution of drug after 60 s for a tube with 3 holes.

To better understand the effect of the size of the hole on the delivery process, simulations with holes of $5$ μm, $10$ μm and $15$ μm of width were computed and the amount of drug infused in the brain along time is plotted [Fig. 4(a)]. The width is not linearly proportional to the amount of drug, in fact increasing the hole size by 2 does not allow twice more drug to diffuse into the brain. Furthermore, increasing the hole size from $5$ to $10$ μm has a bigger impact on the drug release than increasing it from $10$ to 15 μm. A similar behaviour can be observed when varying the number of holes. Indeed, increasing the number of holes to 2 or 3 does not increase as much the amount of drug [Fig. 4(b)]. This is because the drug concentration gradient is shared by the different holes. As the driving transport mechanism in a diffusion-driven delivery is the concentration gradient, this results in a slower delivery. For example, in the case of three holes, even though 3 different circles can be observed at the very beginning of the process [Fig. 4(c)], after a short amount of time, the three circles fuse together forming one big circular distribution [Fig. 4(d)], and thus only one bigger concentration gradient all around the hole instead of three.

## Discussion

When all the elimination processes are suppressed, the amount of drug delivered increases linearly once the process is set (after approx. 1 min). This is because the source of delivery, i.e., the concentration in the tube, stays almost constant with time in the whole tube. The concentration profile, nonetheless, does not increase linearly in time. This can be explained by the drug filling up a 2D circular surface, rather than just a line. Thus, with time, the radius of the concentration of drug distribution increases proportionally to the square root of the total amount of drug, making the increase smaller and smaller as time progresses.

The transport mechanism driving the drug can be either dominated by diffusion or convection. By decreasing the flow rate, the pressure applied on the brain is decreased and as convection is induced by a pressure gradient, this results in a decreasing convection term. By decreasing this term enough, we are able to reach a diffusion-driven process. By looking at the comparison between the diffusive and the convective flux magnitude, it can be observed that, for a small enough flow rate (in this case $3$ nL/min), the diffusive flux magnitude is bigger than the convective flux magnitude [Fig. 2(d)]. This diffusion dominated process can also be observed in the concentration profiles. The diffusion-driven delivery has an exponential-like shape while the convection-driven one has an inverse S-shaped curve, that is, it has a higher concentration extending farther from the delivery site.^[19]^ However, an optimal flow rate must be found, decreasing it too much will result in a higher pressure inside the brain than in the tube thus causing backflow. The optimal flow rate to attain diffusion dominated delivery is contained in a small range and it is dependent on the pressure in the brain but also on the geometry of the tube or the drug delivered.

Different parameters were changed to assess their influence on the delivery process. The first one is the diffusion coefficient, showing us that delivering a bigger molecule as for example a protein, results in a smaller diffusion coefficient and thus, a slower delivery. On the other hand, a smaller molecule, for example an ion (sodium or potassium), has a bigger diffusion coefficient and therefore a faster delivery process. This almost-linear relation between the diffusion coefficient and the total amount of drug delivered arises because of the diffusion-driven regime and could be different with higher flow rates. In the second sweep, it is shown that changing the drug concentration in the tube affects linearly the amount of drug delivered and the latter stays linear in time for any drug concentration. However, integrating an elimination rate in the simulation to take in account the different uptake, loss or clearance processes, has a rather big impact on the shape of the curve. In fact, the amount of drug does not increase linearly any further, but it converges to an equilibrium. This equilibrium is achieved when there is as much drug diffusing out of the tube as there is disappearing from the drug extracellular space. With a higher elimination rate, the amount of drug will converge faster and to a lower value.

Finally, increasing the size of the hole or the number of holes does not increase the drug diffusion linearly. In particular, by having two or three holes, does not result in twice or three times the amount of drug delivered. This is explained by the interaction of the diffusion process between the holes. Indeed, if the holes were far enough from each other, interactions will be avoided and possibly achieve a linear increase in delivery. However, in this case, we can see that instead of having three circular distributions, we have one bigger distribution which produces less concentration gradient and thus, less drug delivered than three independent holes. We posit this phenomenon is similar to the drug flux from a microporous membrane observed with commercially available microdialysis probes^[20–22]^ albeit on smaller length scales. This interaction can be useful as it allows one to achieve a larger and more uniform distribution while keeping a diffusion-driven delivery.

## Conclusion

In this study, drug delivery to the brain extracellular space was simulated for a cellular scale microtube. The aim was to verify the feasibility of diffusion-driven transport while reducing the applied pressure and decreasing the size of the delivery tube. This was achieved by decreasing the width of the tube to $10$ μm and using a U-shaped geometry. In this geometry, most of the drug continues along the tube and is released through the outlet outside of the brain making it possible to avoid almost any pressure on the brain. Thus, by adapting the flow rate, a diffusion-driven delivery was achieved. However, the range of possible flow rates is relatively small and needs to be adapted when changing the size of the tube, the drug or the animal model. It was also shown that the amount of drug delivered increases linearly in time when the elimination process is neglected. Finally, the relationship between the infusion rate, drug-dependent parameters and size or number of holes was examined. Collectively, these findings will accelerate the realization and implementation of diffusion-driven drug delivery technologies.

## Supplementary Information

Below is the link to the electronic supplementary material.

Supplementary file1 (DOCX 15 KB)

## Data Availability

Additional data from this study is accessible in the supporting information.
