# Supplementary material for: Diffusive drug delivery in the brain extracellular space from a cellular scale microtube
Source: MRS Commun. 2022 Sep 19;12(5):654–61. doi: 10.1557/s43579-022-00247-9 (PMC9596563; doi:10.1557/s43579-022-00247-9)
Supplement: Supplementary file 1 — Supplementary file1 (DOCX 15 KB) [file 43579_2022_247_MOESM1_ESM.docx]

**Supporting information:**

Table S1: Parameters for the tube and the brain tissue

| Symbol | Name | Tube | Brain tissue |
| --- | --- | --- | --- |
| $\alpha$ | Porosity $[-]$ | $-$ | $0.2$ |
| $\rho$ | Density $[kg/m^{3}]$ | $1000$ | $1000$ |
| $\mu$ | Dyn. Viscosity $[kg/(m\cdot s)]$ | $8.9\cdot{10}^{-4}$ | $7.8\cdot{10}^{-4}$ |
| $\kappa$ | Permeability $[m^{2}]$ | $-$ | $6.4\cdot{10}^{-15}$ |

Table S2: Parameters for simulation

| Symbol | Name | Value |
| --- | --- | --- |
| FR | Flow rate $[nL/min]$ | $3.0$ |
| ICP | Intracranial pressure [Pa] | $536$ |
| HS | Hole size $[\mu m]$ | $10$ |
| NH | Number of holes $[-]$ | $1$ |
| $D^{*}$ | Diffusion coeff. $[m^{2}/s]$ | ${10}^{-9}$ |
| $C_{d}$ | Drug concentration $[mM]$ | $10$ |
| $k_{elim}$ | Elimination rate $[s^{-1}]$ | $0$ |
